# Total blood lymphocyte counts in hemochromatosis probands with *HFE *C282Y homozygosity: relationship to severity of iron overload and HLA-A and -B alleles and haplotypes

**DOI:** 10.1186/1471-2326-5-5

**Published:** 2005-07-25

**Authors:** James C Barton, Howard W Wiener, Ronald T Acton, Rodney CP Go

**Affiliations:** 1 Southern Iron Disorders Center, Birmingham, Alabama, USA; 2 Department of Medicine, University of Alabama at Birmingham, Birmingham, Alabama, USA; 3 Department of Epidemiology and International Health, University of Alabama at Birmingham, Birmingham, Alabama, USA; 4 Immunogenetics Program, Department of Microbiology, University of Alabama at Birmingham, Birmingham, Alabama, USA

## Abstract

**Background:**

It has been reported that some persons with hemochromatosis have low total blood lymphocyte counts, but the reason for this is unknown.

**Methods:**

We measured total blood lymphocyte counts using an automated blood cell counter in 146 hemochromatosis probands (88 men, 58 women) with *HFE *C282Y homozygosity who were diagnosed in medical care. Univariate and multivariate analyses of total blood lymphocyte counts were evaluated using these variables: sex; age, transferrin saturation, and serum ferritin concentration at diagnosis; units of blood removed by phlebotomy to achieve iron depletion; and human leukocyte antigen (HLA)-A and -B alleles and haplotypes.

**Results:**

The mean age at diagnosis was 49 ± 14 years (range 18 – 80 years) in men and 50 ± 13 years (range 22 – 88 years) in women. The correlations of total blood lymphocyte counts with sex, age, transferrin saturation, and serum ferritin concentration at diagnosis, and units of blood removed by phlebotomy to achieve iron depletion were not significant at the 0.05 level. Univariate analyses revealed significant associations between total blood lymphocyte counts and presence of the HLA-A*01, -B*08, and -B*14 alleles, and the A*01-B*08 haplotype. Presence of the A*01 allele, B*08 allele, or A*01-B*08 haplotype were associated with a lower total blood lymphocyte count, whereas presence of the B*14 allele was associated with a greater total blood lymphocyte count. There was an inverse association of total blood lymphocyte count with units of phlebotomy to achieve iron depletion, serum ferritin concentration, and with presence of the A*01-B*08 haplotype.

**Conclusion:**

We conclude that there is a significant inverse relationship of total blood lymphocyte counts and severity of iron overload in hemochromatosis probands with *HFE *C282Y homozygosity. The presence of the HLA-A*01 allele or the -B*08 allele was also associated with significantly lower total blood lymphocyte counts, whereas presence of the -B*14 allele was associated with significantly higher total blood lymphocyte counts. In univariate and multivariate analyses, total blood lymphocyte counts were significantly lower in probands with the HLA-A*01-B*08 haplotype than in probands without this haplotype.

## Background

Hemochromatosis occurs in 0.003 – 0.005 of persons of northwestern European descent, and is typically associated with homozygosity for the C282Y mutation of the *HFE *gene (exon 2, nt 845 G→A), located ~4 Mb telomeric to the human leukocyte antigen (HLA) region on Ch6p [1,2]. Some persons with hemochromatosis absorb increased quantities of iron and develop severe iron overload that is associated with hepatic cirrhosis, primary liver cancer, diabetes mellitus, other endocrinopathy, arthropathy, and cardiomyopathy, and with reduced longevity [1]. Total blood lymphocyte counts were lower in hemochromatosis index subjects with *HFE *C282Y homozygosity, higher iron stores, and hepatic cirrhosis than in those with lower iron burdens who did not have cirrhosis. In men, there was a significant negative correlation of total blood lymphocyte counts and body iron stores [3].

In the present study, we performed multivariate analyses of the variables sex; age; transferrin saturation and serum ferritin concentration at diagnosis; units of blood removed by phlebotomy to achieve iron depletion; and human leukocyte antigen (HLA)-A and -B alleles and haplotypes to determine their effects on total blood lymphocyte count at diagnosis in hemochromatosis probands with C282Y homozygosity. We discuss the implications of our observations in explaining quantities of total blood lymphocytes previously reported in persons with hemochromatosis associated with C282Y homozygosity.

## Methods

### General criteria for selection of study subjects

The performance of this work was approved by the Institutional Review Boards of Brookwood Medical Center and the University of Alabama at Birmingham. All subjects were adults (≥ 18 years of age) who identified themselves as Caucasians or whites; each resided in central Alabama. All hemochromatosis probands were diagnosed in a single community medical center; none was diagnosed by family or population screening. Probands with diagnoses of primary hematologic malignancies or those receiving anti-cancer chemotherapy were excluded. Persons of African ancestry were excluded for reasons described previously [4-7].

### Selection of hemochromatosis probands

A presumptive diagnosis was established using an elevated transferrin saturation criterion; each proband was evaluated for iron overload and its complications [1,8,9]. We included probands who had: a) diagnosis in medical care during the interval 1997–2002; b) *HFE *C282Y homozygosity; c) available HLA-A and -B haplotypes; and d) therapeutic phlebotomy to induce iron depletion [9]. This cohort is the same as otherwise described and evaluated in a previous study [10].

### Diagnosis of common variable immunodeficiency (CVID) and IgG subclass deficiency (IgGSD)

Diagnoses of CVID or IgGSD were based on demonstration of persistent, otherwise unexplained serum concentrations of Ig >2 SD below the corresponding mean levels [6,7,11]. Criteria for the diagnosis of CVID were: 1) decreased total serum IgG concentration; and 2) either decreased IgG subclass(es), decreased serum IgA concentration, or decreased serum IgM concentration [6,7,11]. Criteria for the diagnosis of IgGSD were: 1) normal total serum IgG concentration; and 2) abnormally low serum concentrations of one or more IgG subclasses; some patients with IgGSD also have decreased levels of IgA or IgM levels, although measurements of IgA or IgM are not diagnostic criteria for CVID or IgGSD [6,7,11].

### Iron-associated measurements

Serum iron concentration, total serum iron-binding capacity, and serum ferritin concentration were measured using automated clinical methods and blood specimens obtained after an overnight fast. Transferrin saturation was expressed as the quotient of serum iron and iron-binding capacity × 100%. In some cases, percutaneous biopsy specimens of liver were obtained as an adjunct to hemochromatosis diagnosis and evaluation of hepatic pathology. Phlebotomy to induce iron depletion was performed as previously described; one unit of phlebotomy was defined as ~500 mL of blood [9]. We used presumptive criteria of iron overload as indications to perform therapeutic phlebotomy: serum ferritin ≥ 300 ng/mL (men) and ≥ 200 ng/mL (women) [9]. Iron overload was defined by demonstration of hepatic iron index ≥ 1.9 or removal of ≥ 2.0 g Fe by therapeutic phlebotomy [12]. Iron depletion was defined as complete when the serum ferritin level was 10 – 20 ng/mL, or when the hemoglobin concentration was <11.0 g/dL or the hematocrit was <33.0% for more than three weeks (in patients without chronic anemia) [9].

### Immunoglobulin measurements

Serum concentrations of IgG, IgG subclasses, IgA, and IgM and were measured using standard automated methods before IgG replacement therapy was initiated, and as nadir values at the time of monthly IgG infusions in some patients. Reference ranges for serum Ig concentrations are: total IgG 700 – 1600 mg/dL; IgG_1 _422 – 1292 mg/dL; IgG_2 _117 – 747 mg/dL; IgG_3 _41 – 129 mg/dL; IgG_4 _1 – 291 mg/dL; total IgA 70 – 400 mg/dL; and IgM 40 – 230 mg/dL. The basis of these reference ranges has been reported elsewhere [13]. Deficiency of an Ig class or subclass was defined by a serum concentration at diagnosis that was less than the corresponding lower reference limit. Quantification of serum concentrations of total serum IgG and IgG subclasses was performed in all hemochromatosis probands. Quantification of IgA and IgM was performed in subjects with CVID and IgGSD, although these analytes were not measured in hemochromatosis probands whose total serum IgG and IgG subclass values were within the corresponding reference ranges. Measurement of IgA subclasses, IgD, or IgE in serum was not routinely performed in any of the present subjects.

### Total blood lymphocyte counts

Blood specimens obtained by antecubital venipuncture from probands at the time of diagnosis of hemochromatosis were analyzed using a Cell-Dyne 1300 automated blood counter (Abbott Laboratories, Chicago, IL). Total blood lymphocyte counts were defined as the numbers of leukocytes of volume 40 – 100 fL detected by the counter in the respective specimens; counts are expressed as cells/mm^3 ^× 10^-3^.

### *HFE *and HLA analyses

*HFE *analyses were performed as described previously [14]. HLA-A and -B alleles were detected using low-resolution DNA-based typing (PCR/sequence-specific oligonucleotide probe) in hemochromatosis probands [14]. Control subjects were tested using the microdroplet lymphocytotoxicity test [15]; subjects were evaluated using antisera that detected allele assignments described in the 9^th ^International Histocompatibility Workshop [16]. Because the levels of resolution of the DNA-based and serological typing methods we used are similar, alleles detected by these respective methods provide concordant allele assignments, with the exception of B*70 and B*71 that were not detected by serological methods. HLA typing of family members permitted assignment of Ch6p haplotypes defined by -A and -B alleles [5,7].

### Statistical considerations

The data set included observations in 146 hemochromatosis probands. Numbers of men and women in various proband subgroups vary because some data were unavailable due to conditions of referral and prior management; we were unable to set phase for HLA haplotype determination in 20 probands. Analyses were performed with SAS [17], a computer spreadsheet (Excel 2000^®^, Microsoft Corp., Redmond, WA), and a statistical program (GB-Stat^® ^v. 10.0, 2003, Dynamic Microsystems, Inc., Silver Spring, MD).

We determined that a log_e _(ln) transformation normalized the iron measures data and total blood lymphocyte counts (expressed as cells/mm^3 ^× 10^-3 ^± 1 SD) [18], and thus permitted the use of statistical techniques that assume that values within a data set are normally distributed. Independent variables included a) sex; b) age at diagnosis; c) transferrin saturation at diagnosis; d) serum ferritin concentration at diagnosis; e) units of blood removed by phlebotomy to achieve iron depletion; and f) HLA-A and -B alleles and haplotypes. Descriptive data are displayed as enumerations, percentages, mean ± 1 S.D. or mean (95% confidence intervals (CI)). Frequency values were compared using chi-square analysis or Fisher exact test (one-tail), as appropriate. Mean values were compared using a student t-test (two-tail). Transformed measures are rounded to two decimal places. Blood lymphocyte counts were expressed to the nearest one decimal place. Frequencies and p values are expressed to four significant figures. We used an algorithm applicable to loci with multiple alleles [19] to estimate the significance level of Hardy-Weinberg proportions of HLA-A and -B allele frequencies in hemochromatosis probands. Two sets of analysis of variance (ANOVA) models were fit to the log_e_-transformed total blood lymphocyte count data. The first set used indicators of single HLA-A and -B alleles, and the second used HLA-A and -B haplotypes. The overall fits of the ANOVA models are indicated by R^2 ^values; values of p < 0.05 were defined as significant.

## Results

### General characteristics of hemochromatosis probands with *HFE *C282Y homozygosity

There were 146 probands (88 men, 58 women). The mean age at diagnosis was 49 ± 14 years (range 18 – 80 years) in men and 50 ± 13 years (range 22 – 88 years) in women. Iron measures are displayed in Table 1. Eighty-six men and 42 women had iron overload. Fifteen men and six women had hepatic cirrhosis proven by liver biopsy (14.4%). Nine men and two women reported that they consumed ≥ 60 g of ethanol daily (7.5%). Three men had chronic hepatitis C; one man had porphyria cutanea tarda. None had undergone splenectomy, and none had lymphoproliferative disorders. Thirteen probands had either CVID (n = 3) or IgGSD (n = 10) (7 men, 6 women).

**Table 1:** Iron measures in hemochromatosis probands with *HFE *C282Y homozygosity^1^

Iron measure	Men (95% CI) [*n*]	Women (95% CI) [n]	p value
Serum iron, μg/dL	209 (137, 317) [*81*]	193 (118, 315) [*51*]	0.0523
Transferrin saturation, %	85 (59, 121) [*83*]	77 (44, 133) [*52*]	0.0120
Serum ferritin, ng/mL	1097 (209, 5768) [*81*]	546 (84, 3535) [*56*]	<0.0001
Phlebotomy to achieve iron depletion, units	29 (7, 122) [*73*]	17 (4, 76) [*42*]	0.0001

^1^Serum iron, transferrin saturation, and serum ferritin levels were measured at diagnosis of hemochromatosis. Values were transformed (log_e_) to achieve normal distributions; comparisons were made using a student t-test (two-tail). Data displayed in the table are expressed as mean (95% CI) after computing antilogs_e _of the transformed data; p values < 0.05 were defined as significant.

Mean transferrin saturation, mean serum ferritin concentration, and mean units of phlebotomy to achieve iron depletion were greater in men than women with hemochromatosis (Table 1). The mean units of phlebotomy to achieve iron depletion was approximately twice as great in men as women (Table 1).

### Hardy-Weinberg proportions of HLA-A and HLA-B alleles

Frequencies of HLA-A and -B alleles in hemochromatosis probands did not depart significantly from Hardy-Weinberg equilibrium.

### Overall frequencies of HLA-A*03 allele and HLA-A and -B haplotypes

The frequencies of HLA-A*03 in male and female hemochromatosis probands were similar (0.8023 *vs. *0.6727; p = 0.0823). Frequencies of HLA-A*03 in male and female control subjects were also similar (0.2662 *vs. *0.2815; p = 0.7162) [10]. The overall frequency of A*03 in hemochromatosis probands was greater than that in control subjects (0.7518 *vs. *0.2787; p < 0.0001).

Frequencies of the most common haplotypes detected in *HFE *C282Y homozygotes with a hemochromatosis phenotype from this geographic area [5,6,10] were compared with corresponding frequencies in control subjects. The overall frequency of A*01-B*08 was lower in hemochromatosis probands than in control subjects, but the difference was not significant (0.0603 *vs. *0.0927, respectively; p = 0.0634). The overall frequency of A*02-B*44 was similar in hemochromatosis probands and in control subjects (0.0461 *vs. *0.0620, respectively; p = 0.2846). The overall frequency of A*03-B*07 was greater in hemochromatosis probands than in control subjects (0.2447 *vs. *0.0520, respectively; p < 0.0001, respectively). The overall frequency of A*03-B*14 was greater in hemochromatosis probands than in control subjects (0.0709 *vs. *0.0113, respectively; p < 0.0001).

### Comparison of log_e _total blood lymphocyte counts in men and women

Univariate analyses of mean total blood lymphocyte counts in men and women were expressed as cells/mm^3 ^× 10^-3 ^(95% CI). In men, the mean was 1.9/mm^3 ^× 10^-3 ^(1.1, 3.5). In women, the mean was 2.0/mm^3 ^× 10^-3 ^(1.1, 3.5). These values were not significantly different (p = 0.2473).

### Correlation of log_e _total blood lymphocyte counts with clinical variables

The correlations of log_e _total blood lymphocyte counts with sex; age, log_e _transferrin saturation, log_e _serum ferritin concentration at diagnosis; and log_e _units of blood removed by phlebotomy to achieve iron depletion were not significant at the 0.05 level.

### Univariate analyses of log_e _total blood lymphocyte counts and HLA-A and -B alleles

Univariate analyses revealed significant associations between mean log_e _total blood lymphocyte count and presence of the HLA-A*01, -B*08, and -B*14 alleles, and the A*01-B*08 haplotype (Table 2). Presence of the -A*01 allele, -B*08 allele, or A*01-B*08 haplotype was associated with lower total blood lymphocyte counts, whereas presence of the -B*14 allele was associated with greater total blood lymphocyte counts.

**Table 2:** Mean total blood lymphocyte counts in hemochromatosis probands with *HFE *C282Y homozygosity

HLA	log_e _lymphocyte count (cells/mm^3 ^× 10^-3 ^(SD))	lymphocyte count (cells/mm^3 ^× 10^-3 ^(95% CI))^1^	p value (present *vs. *absent)^2^
A*01 present	1.012 (0.213)	2.8 (1.8, 4.2)	0.0362
A*01 absent	1.101 (0.184)	3.0 (2.1, 4.3)	
B*08 present	0.975 (0.178)	2.7 (1.9, 3.8)	0.0048
B*08 absent	1.104 (0.189)	3.0 (2.1, 4.4)	
B*14 present	1.160 (0.180)	3.2 (2.2, 4.5)	0.0317
B*14 absent	1.065 (0.192)	2.9 (2.0, 4.2)	
A*01-B*08 present	0.943 (0.195)	2.6 (1.8, 3.8)	0.0026
A*01-B*08 absent	1.101 (0.185)	3.0 (2.1, 4.3)	

^1^Lymphocyte counts were measured at diagnosis of hemochromatosis, and are expressed as mean (95% CI) after computing antilogs_e _of the transformed data.

^2^Comparisons were made using log_e _lymphocyte counts and student t-test (two-tail); p values < 0.05 were defined as significant.

### Multivariate analyses of log_e _total blood lymphocyte counts and clinical variables, HLA-A and -B alleles, and HLA-A and -B haplotypes

The residual variance formed by accounting for age and gender effects were used to explore multivariable associations. The HLA alleles that predicted log_e_ total blood lymphocyte counts were B*08 (p = 0.0283) and B*14 (p = 0.0204). The presence of B*08 was associated with lower log_e_ total blood lymphocyte counts, whereas the presence of B*14 was associated with higher log_e_ total blood lymphocyte counts. When the residuals of the other clinical variables were added to the model, the effects of units of phlebotomy to induce iron depletion, serum ferritin concentration, and B*08 were significant (p = 0.0326, 0.0172, and 0.0127, respectively). Mean lymphocyte counts were lower with increasing serum ferritin concentration.

Similar models were fit using the HLA-A and -B haplotypes as variables. The A*01-B*08 haplotype was the only significant predictor of total blood lymphocyte count (p = 0.0021), and the presence of A*01-B*08 was associated with lower total blood lymphocyte counts after accounting for age and gender. When other variables were added to this same model, the units of phlebotomy to induce iron depletion (p = 0.0440), serum ferritin concentration (p = 0.0108) and the effect of A*01-B*08 (p = 0.0023) remained significant. A decrease in log_e _total blood lymphocyte count was associated with an increase in units of phlebotomy to induce iron depletion, log_e _serum ferritin concentration, and with presence of the A*01-B*08 haplotype.

### Univariate analyses of log_e _total blood lymphocyte counts and hepatic cirrhosis

The mean total blood lymphocyte count in the 21 probands with hepatic cirrhosis proven by biopsy (1.9 cells/mm^3 ^× 10^-3 ^(95% CI: 1.1 × 10^-3^, 3.3 × 10^-3^)) was similar to that in the 125 probands without cirrhosis (1.9 cells/mm^3 ^× 10^-3 ^(95% CI: 1.1 × 10^-3^, 3.5 × 10^-3^); p = 0.8514).

### Univariate analyses of log_e _total blood lymphocyte counts and CVID or IgGSD

The mean total blood lymphocyte count in the 13 probands with CVID or IgGSD (2.0 cells/mm^3 ^× 10^-3 ^(95% CI: 1.5 × 10^-3^, 2.6 × 10^-3^)) was similar to that in the 133 probands who did not have CVID or IgGSD (1.9 cells/mm^3 ^× 10^-3 ^(95% CI: 1.0 × 10^-3^, 3.5 × 10^-3^); p = 0.5814).

## Discussion

The present study is comprised of the largest number of *HFE *C282Y homozygotes with hemochromatosis phenotypes who had available HLA-A and -B allele and haplotype data and were evaluated for the effects of clinical variables on total blood lymphocyte counts. Overall, the mean total blood lymphocyte counts in the present 88 male and 58 female hemochromatosis probands are very similar to those determined by automated methods in 100 male and 100 female healthy volunteer Caucasians [20]. Taken together, these results confirm observations of Cruz *et al. *that total blood lymphocyte counts of C282Y homozygotes with hemochromatosis phenotypes (37 men, 9 women) did not differ significantly from those of unrelated normal control subjects (116 men, 148 women) [21] and those of Porto *et al. *that there is an association between low CD8(+) numbers, HLA phenotype, and severity of iron overload [22].

In a multivariate analysis, we observed that total blood lymphocyte counts were lower in probands who required greater numbers of units of phlebotomy to achieve iron depletion or who had greater serum ferritin concentrations at diagnosis. This is consistent with a previous report of a significant inverse correlation of total blood lymphocyte counts with iron stores quantified by phlebotomy in a smaller hemochromatosis case series [3]. Although measurement of blood lymphocyte subsets was beyond the scope of the present study, it has been reported that proportions of the two major peripheral T-lymphocyte subsets expressed as CD4/CD8 ratio are stable before and after phlebotomy therapy for hemochromatosis, confirming the existence of a homeostatic mechanism that regulates the relative numbers of the two major blood T-lymphocyte populations [22,23]. Before the discovery of *HFE*, it was reported that inheritance of part or all of the hemochromatosis ancestral haplotype that includes HLA-A*03 and -B*07, particularly in a homozygous configuration, was associated with evidence of more severe iron overload in hemochromatosis patients in Australia, Alabama, and Italy [24-26]. Further, Porto *et al. *demonstrated that the severity of iron overload quantified by phlebotomy in patients with hemochromatosis was correlated with the proportions of CD4(+) and CD8(+) blood lymphocytes and the presence or absence of HLA-A*03 [22]. Thus, the latter report integrated then-existing knowledge of the relationships of severity of iron overload, HLA types, and blood lymphocyte subsets in persons with hemochromatosis.

Some observations support the hypothesis that lymphocyte numbers could influence iron absorption and therefore severity of iron overload in hemochromatosis. High CD4/CD8 ratios appear to precede the development of severe iron overload in persons with hemochromatosis [23,27]. Persons with hemochromatosis have significantly different CD(8)+ blood lymphocyte subsets than normal control subjects, based on analysis of CD(28) positivity or negativity [28]. In mice, blood lymphocyte numbers may influence iron overload severity in the absence of functional HFE protein [29]. There is a candidate mechanism that could account for a lymphocyte-mediated influence on iron absorption and severity of iron overload in hemochromatosis. Interleukin-6 (IL-6), a cytokine produced predominantly by lymphocytes and macrophages [30,31], induces expression of hepcidin, a potent inhibitor of iron absorption [32]. Further, hepcidin levels are significantly decreased in persons who have hemochromatosis associated with mutations of *HFE *(Ch6p21.3) [32]. Although most reports of lymphocyte numbers and subsets have been made in persons presumed or documented to have HLA- or *HFE*-associated hemochromatosis or iron overload, lymphopenia also occurred in an unusual case of early age-of-onset hemochromatosis and severe iron overload associated with homozygosity for a hepcidin promoter mutation on Ch19q13 [33].

Some reports indicate that lymphocyte numbers do not influence iron absorption either in patients with hemochromatosis or in those with iron overload due to other causes. In the present study, we observed that the mean blood lymphocyte counts were similar in men and women. In an earlier study of the same cohort, we observed that the severity of iron overload was significantly greater in men than women [10]. However, there was significant disparity in the frequency of certain HLA-A and -B types and haplotypes between men and women, but there was no significant association of these HLA markers with the severity of iron overload in a multivariate analysis that included sex as a independent variable [10]. Hepcidin levels are significantly decreased in hemochromatosis associated with *TFR2*, *FPN1*, and *HJV *mutations [34-36]. However, it is unknown whether there is an inverse association of blood lymphocyte numbers and the severity of iron overload or whether lymphocytes contribute to decreased hepcidin levels in these disorders. In patients with beta-thalassemia major, there was a highly significant linear increase in the percentages of blood OKT8(+) cells with an increasing number of units of erythrocytes transfused, irrespective of splenectomy [37]. The percentage of blood OKT4(+) cells varied inversely with increasing numbers of units of erythrocyte transfusion in patients who had not undergone splenectomy; in those who had undergone splenectomy, no significant correlation was observed [37]. Inverse relationships of CD8(+) blood lymphocytes and severity of transfusion iron overload were also observed in persons with beta-thalassemia, and deferoxamine therapy was associated with an increase in CD8(+) blood lymphocytes [38]. In sub-Saharan Africans with African iron overload, a disorder that is typically not linked to HLA or *HFE *C282Y, there was no significant association of serum ferritin concentrations and total blood lymphocyte counts [3,39]. In an experimental model of secondary iron overload in rats, the distribution of lymphocyte subsets in blood, thymus, spleen, mesenteric lymph nodes, Peyer patches, and bone marrow were similar in control and experimental groups [40]. Altogether, these results suggest that there is not a consistent relationship of severity of iron overload with CD4/CD8 ratios, blood T-lymphocyte subsets, or abnormal total blood lymphocyte counts in patients with hemochromatosis or in those with iron overload due to other causes [3,41].

A putative gene on Ch6p that modifies iron overload severity in hemochromatosis is presumed to be linked predominantly to A*03 or A*03-B*07 [14,22,24-26,42]. At present, there are two candidate genes. One is localized to the region of D6S105 [42]. The multivariate analysis of a large cohort of hemochromatosis probands with *HFE *C282Y homozygosity demonstrated that A*03-B*07 has no significant effect on units of phlebotomy to achieve iron depletion, but did not exclude a putative modifier gene in this region [10]. In another study, extended haplotypes of the Ch6p21.3 region in hemochromatosis patients and their "phenotypically unaffected" relatives with *HFE *C282Y homozygosity were similar [43]. Another candidate is tumor necrosis factor (TNF)-α promoter polymorphisms [44]. In an independent case series, however, a positive relationship of TNF-α promoter polymorphisms with iron overload severity or its complications was not confirmed [45]. Altogether, these later observations do not strongly support previous hypotheses that putative genes or alleles on Ch6p modify the severity of iron overload in C282Y homozygotes with hemochromatosis.

Our data set permitted an analysis of the relationship of total blood lymphocyte counts and HLA-A and -B alleles and haplotypes. In the present hemochromatosis probands, univariate analyses revealed that the presence of the HLA-A*01 allele or the -B*08 allele was associated with lower total blood lymphocyte counts, whereas presence of the -B*14 allele was associated with greater total blood lymphocyte counts. Bryan *et al. *first suggested a possible role for HLA in the interaction of iron, HLA, and lymphocytes by demonstrating that there was a differential response of peripheral blood mononuclear cells from HLA-A*02 and non-HLA-A*02 donors when the respective lymphocyte isolates were exposed to iron in a mixed lymphocyte culture reaction [46]. In hemochromatosis families and random population control subjects from Portugal, significantly higher blood CD8(+) lymphocyte counts were observed in subjects who had both the *HFE *H63D mutation and the HLA-A*29 allele [47]. In a control population from Portugal, there was a significant correlation of the HLA-A*01 with high numbers of CD8(+) blood lymphocytes, and an association of HLA-A*24 with low numbers of CD8(+) blood lymphocytes [21]. These observations indicate that total blood lymphocyte counts or blood T-lymphocyte subsets in persons who inherit common *HFE *missense mutations (with or without hemochromatosis) are associated with HLA-A and -B alleles.

In the present hemochromatosis probands, A*01-B*08 was a significant predictor of lower total blood lymphocyte counts in univariate and multivariate analyses. Further, A*01-B*08 is associated with greater serum ferritin concentrations in older hemochromatosis probands with C282Y homozygosity grouped by age than other haplotypes [10]. However, the association of A*01-B*08 and the severity of iron overload quantified by phlebotomy to achieve iron depletion was not significant [10]. In persons without hemochromatosis, total blood lymphocyte counts are lower in those with HLA-A*01-B*08, DR3 than in persons with other HLA haplotypes [48,49]. Persons with human immunodeficiency virus (HIV) infections and HLA-A*01-B*08 have lower total blood lymphocyte counts than persons with HIV infections who do not have HLA-A*01-B*08 [50,51]. Taken together, these observations suggest that there is a determinant of total blood lymphocyte counts or CD8(+) blood lymphocyte counts within the HLA-A*01-B*08 haplotype or in linkage disequilibrium with it. These observations also support previous reports that genetic factors in the region of the major histocompatibility complex on chromosome 6 have a major influence on the variation in blood lymphocyte numbers, especially those of T-lymphocyte subsets, in humans [21,52,53]. In a study of 15 CEPH families, quantitative trait loci that accounted for significant proportions of the phenotypic variance of blood lymphocyte counts and blood lymphocyte subpopulations were also detected on chromosomes 1, 2, 3, 4, 8, 9, 11, 12, and 18 [54].

In the present study, there was no significant difference in the total blood lymphocyte counts of hemochromatosis probands with or without hepatic cirrhosis in a univariate analysis. In contrast, it has been reported that total blood lymphocyte counts were lower in hemochromatosis index subjects with C282Y homozygosity with hepatic cirrhosis than in those with lower iron burdens who did not have cirrhosis [3]. However, the latter investigators indicated that their overall findings argue against the possibility that low blood lymphocyte counts in *HFE *hemochromatosis are a consequence of iron overload or represent an epiphenomenon of advanced cirrhosis [3,41]. The differences in the results of the present study and those of a previous report [3] may also be due to ethnic differences in the respective study populations, and the greater number of patients and lower prevalence of hepatic cirrhosis in the present report (n = 146; 14% had cirrhosis *vs. *previous report: n = 20; 65% had cirrhosis).

Total blood lymphocyte counts, including T-and B-lymphocyte subset counts, are subnormal in some persons with CVID [55,56]. However, we did not detect a significant difference in the total blood lymphocyte counts in hemochromatosis probands with or without CVID or IgGSD. This is consistent with the generally less severe blood lymphocyte subset deficits in IgGSD than in CVID [55,57], and with the greater proportion of the hemochromatosis probands in the present and another cohort who had IgGSD than CVID [6].

## Conclusion

We conclude that there is a significant inverse relationship of total blood lymphocyte counts and severity of iron overload in hemochromatosis probands with *HFE *C282Y homozygosity. The presence of the HLA-A*01 allele or the -B*08 allele was also associated with significantly lower total blood lymphocyte counts, whereas presence of the -B*14 allele was associated with significantly higher total blood lymphocyte counts. In univariate and multivariate analyses, total blood lymphocyte counts were significantly lower in probands with the HLA-A*01-B*08 haplotype than in probands without this haplotype.

## Competing interests

The author(s) declare that they have no competing interests.

## Authors' contributions

JCB conceived and designed the study, diagnosed and treated the hemochromatosis probands and compiled their clinical data, performed some of the statistical analyses, and contributed to writing the manuscript. HWW performed statistical analyses and contributed to writing the manuscript. RTA compiled data on hemochromatosis probands, performed HLA and *HFE *typing of many of the probands, performed HLA typing of all control subjects, and contributed to writing the manuscript. RCP contributed to statistical analyses and writing the manuscript. All authors approved of the manuscript in its final form.

## Pre-publication history

The pre-publication history for this paper can be accessed here:


